# Laparoscopic versus Open Peritoneal Dialysis Catheter Insertion: A Meta-Analysis

**DOI:** 10.1371/journal.pone.0056351

**Published:** 2013-02-15

**Authors:** Sander M. Hagen, Jeffrey A. Lafranca, Ewout W. Steyerberg, Jan N. M. IJzermans, Frank J. M. F. Dor

**Affiliations:** 1 Department of Surgery, Erasmus MC, University Medical Center, Rotterdam, The Netherlands; 2 Department of Public Health, Erasmus MC, University Medical Center, Rotterdam, The Netherlands; University of Colorado, United States of America

## Abstract

**Background:**

Peritoneal dialysis is an effective treatment for end-stage renal disease. Key to successful peritoneal dialysis is a well-functioning catheter. The different insertion techniques may be of great importance. Mostly, the standard operative approach is the open technique; however, laparoscopic insertion is increasingly popular. Catheter malfunction is reported up to 35% for the open technique and up to 13% for the laparoscopic technique. However, evidence is lacking to definitely conclude that the laparoscopic approach is to be preferred. This review and meta-analysis was carried out to investigate if one of the techniques is superior to the other.

**Methods:**

Comprehensive searches were conducted in MEDLINE, Embase and CENTRAL (the Cochrane Library 2012, issue 10). Reference lists were searched manually. The methodology was in accordance with the Cochrane Handbook for interventional systematic reviews, and written based on the PRISMA-statement.

**Results:**

Three randomized controlled trials and eight cohort studies were identified. Nine postoperative outcome measures were meta-analyzed; of these, seven were not different between operation techniques. Based on the meta-analysis, the proportion of migrating catheters was lower (odds ratio (OR) 0.21, confidence interval (CI) 0.07 to 0.63; P = 0.006), and the one-year catheter survival was higher in the laparoscopic group (OR 3.93, CI 1.80 to 8.57; P = 0.0006).

**Conclusions:**

Based on these results there is some evidence in favour of the laparoscopic insertion technique for having a higher one-year catheter survival and less migration, which would be clinically relevant.

## Introduction

Peritoneal dialysis (PD) is an effective treatment for end-stage renal disease (ESRD) [1]–[4]. The most important benefit of PD relative to haemodialysis is the preservation of residual renal function, which equates to improved survival during the first several years of therapy [5]. The key to successful PD is the presence of a well-functioning dialysis catheter, defined as one that facilitates free dialysis solution in- and outflow. However, several complications, such as in- and outflow obstruction, peritonitis, exit-site infections, leakage and migration, can lead to catheter removal and loss of peritoneal access [6]. Currently, different surgical techniques are in practice for PD catheter placement [6]–[10]. The insertion technique may have a great influence on the occurrence of complications. The literature describes a 10–35% catheter failure rate when using the open technique [11]–[14] and 2.8–13% catheter failures for the laparoscopic insertion technique [15]–[18].

The open technique is still the most frequently used technique. However, laparoscopic procedures have proven to be superior to a number of open surgical procedures, by reducing morbidity, length of hospital stay, postoperative pain and lead to a quicker convalescence [19]–[22]. In case of PD catheter insertion, the laparoscopic approach enables the surgeon to insert the PD catheter under direct vision and thus at the end of the operation the correct catheter position is assured, which may lead to a better and prolonged catheter function.

In the existing literature, there is no consensus about the preferred operative technique for PD catheter insertion. Our aim is to investigate whether there is a preferable method or not, when data from the literature are reviewed and analyzed systematically. In 2004, Strippoli et al. [23] performed a review of the literature up to April 2004, summarizing data comparing laparoscopic, peritoneoscopic and open insertion of PD catheters. This study only included randomized controlled trials and the primary outcome was the prevention of peritonitis. Furthermore, in 2012, Xie et al. [24] performed a review and meta-analysis of the literature. However, this study also included trials using other techniques and studying other populations. Our systematic review includes randomized controlled trials as well as cohort studies up to October 2012, describing multiple outcomes of studies comparing the laparoscopic and open technique in adults.

## Methods

All aspects of the Cochrane Handbook for Interventional Systematic Reviews were followed and the study was written according to the Preferred Reporting Items for Systematic Reviews and Meta-Analyses (PRISMA) statement [25]. A review protocol was drafted before the initial search was started.

### Literature Search Strategy

Comprehensive searches were carried out in MEDLINE, Embase and CENTRAL (the Cochrane Library 2012, issue 10). The search was performed for articles published up to October 2012 relevant to outcome of laparoscopic or open insertion of a PD catheter. There was no publication year or publication language restriction applied. The search-string used in PubMed was (“Peritoneal Dialysis”[Majr] AND (Laparoscopy OR laparotomy OR open)) AND (“catheters”[Majr] OR catheter). Other databases were searched with comparable terms, suitable for the specific database. Reference lists of the identified relevant studies were scrutinized for additional citations.

### Literature Screening

Studies were evaluated for inclusion by two independent researchers (SMH, JAL) for relevance to the subject. A random check was performed by a supervisor (FJMFD). Study selection was accomplished through three phases of study screening. In phase 1, the following types of studies were excluded: reviews, case-reports, letters, editorials, case-series, and papers studying non-human, infants and/or adolescents. In phase 2, abstracts were reviewed for relevance and the full-text articles were obtained. In phase 3, full-text articles were reviewed; inclusion required studies describing laparoscopic and open insertion of the PD catheter. The studies had to describe one or more of the following outcome measures to be included: incidence of peritonitis, exit-site/tunnel infection, pericanullar leakage, catheter migration, catheter removal for complications, need for revision and catheter survival. Any discrepancies in in- or exclusion were resolved by discussion between the reviewers with supervision of a third person.

### Data Extraction and Critical Appraisal

The level of evidence of each paper was established following the Oxford Centre for Evidence-Based Medicine Level of Evidence scale [26], [27] and by using the GRADE tool [28]. The quality and the potential of bias of the randomized controlled trials were assessed according to the Cochrane Collaboration’s tool for assessing risk of bias by Higgins [29].

### Statistical Analysis

Odds ratios (OR) and their 95% confidence interval (CI) were calculated from raw data using patients with an open catheter insertion as the control group. A meta-analysis was performed with complications and catheter survival as outcome measures using Review Manager Software (RevMan, 5.1; The Nordic Cochrane Centre, Copenhagen, Denmark). Each study was weighted by sample size. Heterogeneity of treatment effects between studies was tested using the Q (heterogeneity χ^2^) and the I^2^ statistics. A random-effects model was used for calculating the summary estimates and 95% CI, to account for possible clinical heterogeneity. Overall effects were determined using the Z-test. In addition, the individual study effect on the results was examined by removing each study at a time to examine whether removing a particular study would significantly change the results.

## Results

**Figure 1:**
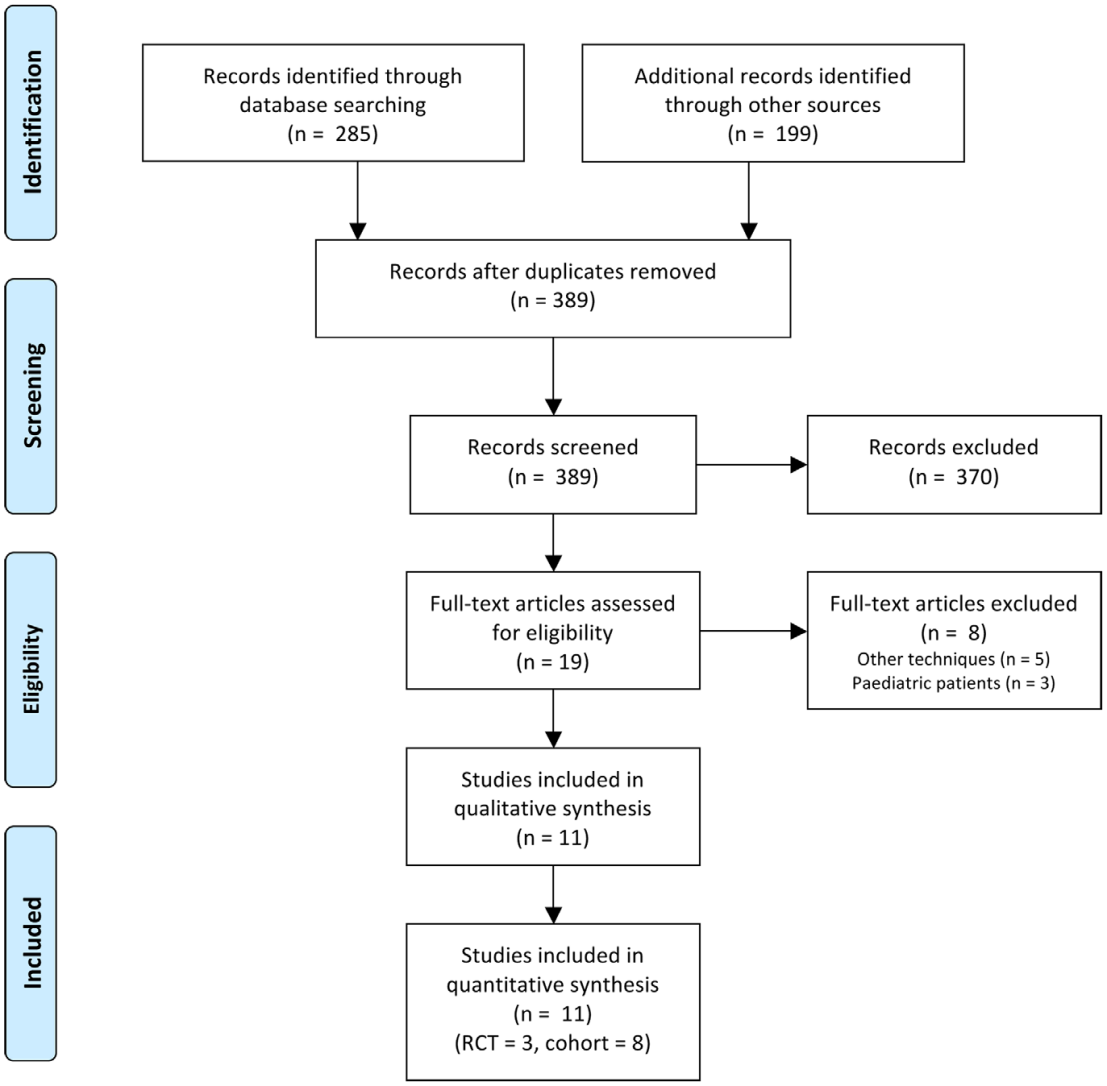
PRISMA flow diagram of the systematic literature search.

**Figure 2:**
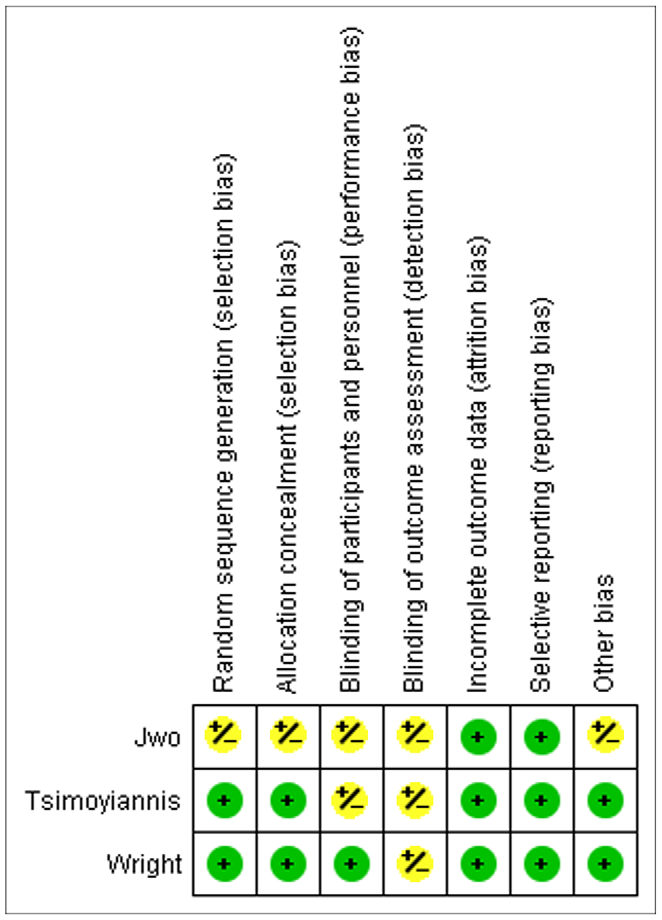
Risk of bias summary graph of the included studies. The green symbol indicates that there is possibly a low level of bias, red symbolizes a possible high level of bias and a yellow symbol is presented if the risk of bias is unclear.

Of the 285 papers found after the initial search, eleven fell within the scope of the study; three randomized controlled trials [14], [30], [31] and eight cohort studies [32]–[39]. These eleven studies were represented by twelve individual references. One publication (by Crabtree et al. 2005) was excluded for describing patients that were already described in another paper in 2000 by the same group [40]. No additional studies were included after manually scrutinizing reference lists. The PRISMA [25] flow diagram for systematic reviews is presented in figure 1. The assessment of the quality of the included studies is presented in figure 2. A meta-analysis was performed using a total of eleven studies; the characteristics of these studies are presented in table 1. Definitions of the analyzed outcome measures are presented in table 2.

**Table 1:** Characteristics of studies comparing laparoscopic and open PD catheter insertion.

Reference	Year	Country	Study type	Groups	*N*	Evidence
Li [39]	2011	Taiwan	Prospective cohort	Laparoscopic	50	2b
				Open	23	
Jwo [14]	2010	Taiwan	RCT	Laparoscopic	37	2b
				Open	40	
Gajjar [32]	2007	USA	Retrospective cohort	Laparoscopic	45	2b
				Open	30	
Lund [38]	2007	Denmark	Retrospective cohort	Laparoscopic	9	2b
				Open	13	
Crabtree [33]	2005	USA	Prospective cohort	Laparoscopic	278	2b
				Open	63	
Soontrapornchai [34]	2005	Thailand	Prospective cohort	Laparoscopic	50	2b
				Open	52	
Ögünç [35]	2003	Turkey	Prospective cohort	Laparoscopic	21	2b
				Open	21	
Batey [37]	2002	USA	Retrospective cohort	Laparoscopic	14	2b
				Open	12	
Tsimoyiannis [30]	2000	Greece	RCT	Laparoscopic	25	2b
				Open	25	
Wright [31]	1999	UK	RCT	Laparoscopic	24	1b
				Open	21	
Draganic [36]	1998	Australia	Retrospective cohort	Laparoscopic	30	2b
				Open	30	

RCT: Randomized controlled trial, n.a.: not applicable.

**Table 2:** List of variables/outcome measures meta-analyzed and the definitions stated by the authors.

Reference	Peritonitis	Exit-site Infection	Migration	Catheter obstruction	Leakage	Intervention/revision	Catheter removal	Catheter survival
Li [39]	Abdominal pain,cloudy effluent with whiteblood cell count higherthan 100/mm3 and/or polymorphonuclear neutrophils larger than 50%, and identification of microorganisms	Not described	transient or prolonged, catheter malfunction during follow up and confirmed by abdominal KUB films	–	Fluid extravasation from the catheterexit-site related to dialysate infusion	–	–	‘catheter dysfunction-free’
Jwo [14]	Not described	Not described	Not described	–	exit-site leak, wound leak, or extra-abdominaldialysate outflow	–	Inadequate dialysis, peritonitis, hydrocele, hydrothorax, change to HD	catheter survival excluding patients with catheter dropout due to clearly unrelated causes such as renal transplantation, renal recovery, or death from unrelated underlying diseases
Tsimoyiannis [30]	Not described	–	Not described	–	Not described	–	Peritonitis	Not described
Wright [31]	Early: <6 weeks post-op Late: >6 weeks post-op	Not described	–	Not described	Not described	–	(pseudomonas) peritonitis, ultra filtration failure, patient death	See ‘removal’
Gajjar [32]	Not described	Not described	–	Not described	Early: occur within30 days of insertion,Late: occur after 30days of insertion	Not described	–	–
Crabtree [33]	–	–	–	Not described	Not described	–	–	–
Soontrapornchai [34]	Not described	Not described	Radiological confirmation	Not described	Not described	–	–	calculated from the day of insertion to the day of revision or removal
Ögünç [35]	Early: <4 weeks post-op Late: >4 weeks post-op	Positive microbiological ofan organism from peritoneal fluid on ether Gram stainingor culture	–	Due to omental wrapping and/orfibrin clotting	Not described	Pericatheter leak,chronic tunnel infection,chronic exit-siteinfection	Relapse or resistant peritonitis, Successful transplantation, Persistent dialysate leak, exit-site infection, patient’s choice, treatment failure	‘Catheter failure free’
Draganic [36]	positive microbiological identification of an organism from peritoneal fluid on either Gram staining or culture	any surrounding inflammation which required additional dressings or antibiotics	–	Not described	–	–	Exit-site infection, peritonitis	–
Batey [37]	–	–	–	–	Not described	Migration, Occlusion	Peritonitis, infected hematoma, oersistent scrotal swelling	–
Lund [38]	Not described	–	Not described	–	–	‘Displacement’	‘Displacement’	–

KUB = kidneys, ureters and bladder.

### Infections (Peritonitis, Exit-site/Tunnel Infection)

Nine studies [14], [30]–[32], [34]–[36], [38], [39] that investigated the incidence of peritonitis after PD catheter insertion were included for meta-analysis, with a total of 541 patients. There was no statistically significant difference in the risk of developing peritonitis between treatment groups (OR 0.83, 95% CI 0.48 to 1.42; P = 0.49).

**Figure 3:**
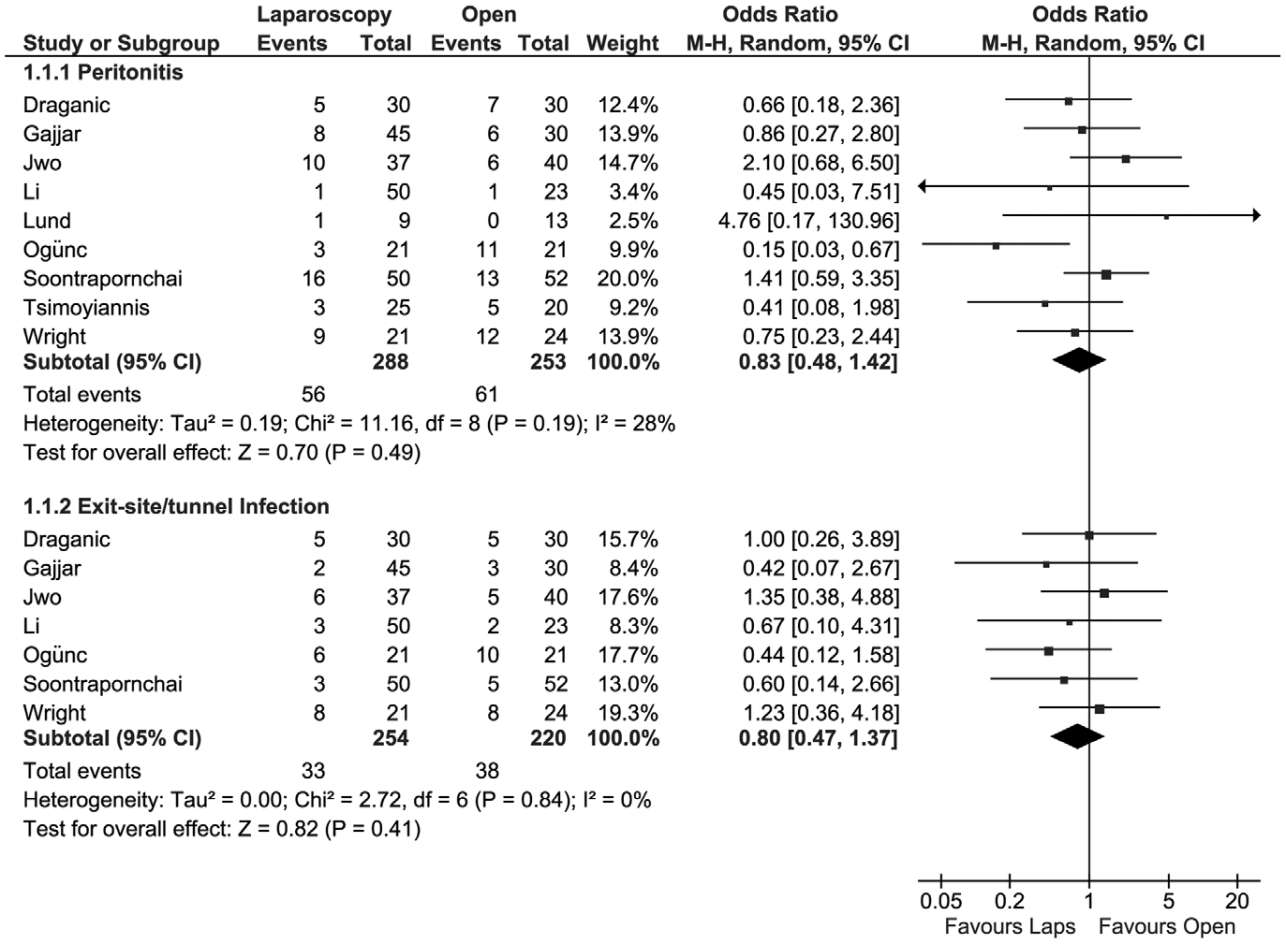
Forest plot. Odds ratios of the incidence of peritonitis and exit-site/tunnel infection, evaluating the statistical difference between laparoscopic and open PD catheter insertion. CI: confidence interval.

With a total of 474 patients from seven studies [14], [31], [32], [34]–[36], [39], the pooled incidence of exit-site/tunnel infection was calculated in the meta-analysis. There was no statistically significant difference in the risk of developing an exit-site/tunnel infection between laparoscopic or open PD catheter insertion (OR 0.80, 95% CI 0.47 to 1.37; P = 0.41). (figure 3).

### Catheter-related Outcome (Migration, Leakage and Obstruction)

**Figure 4:**
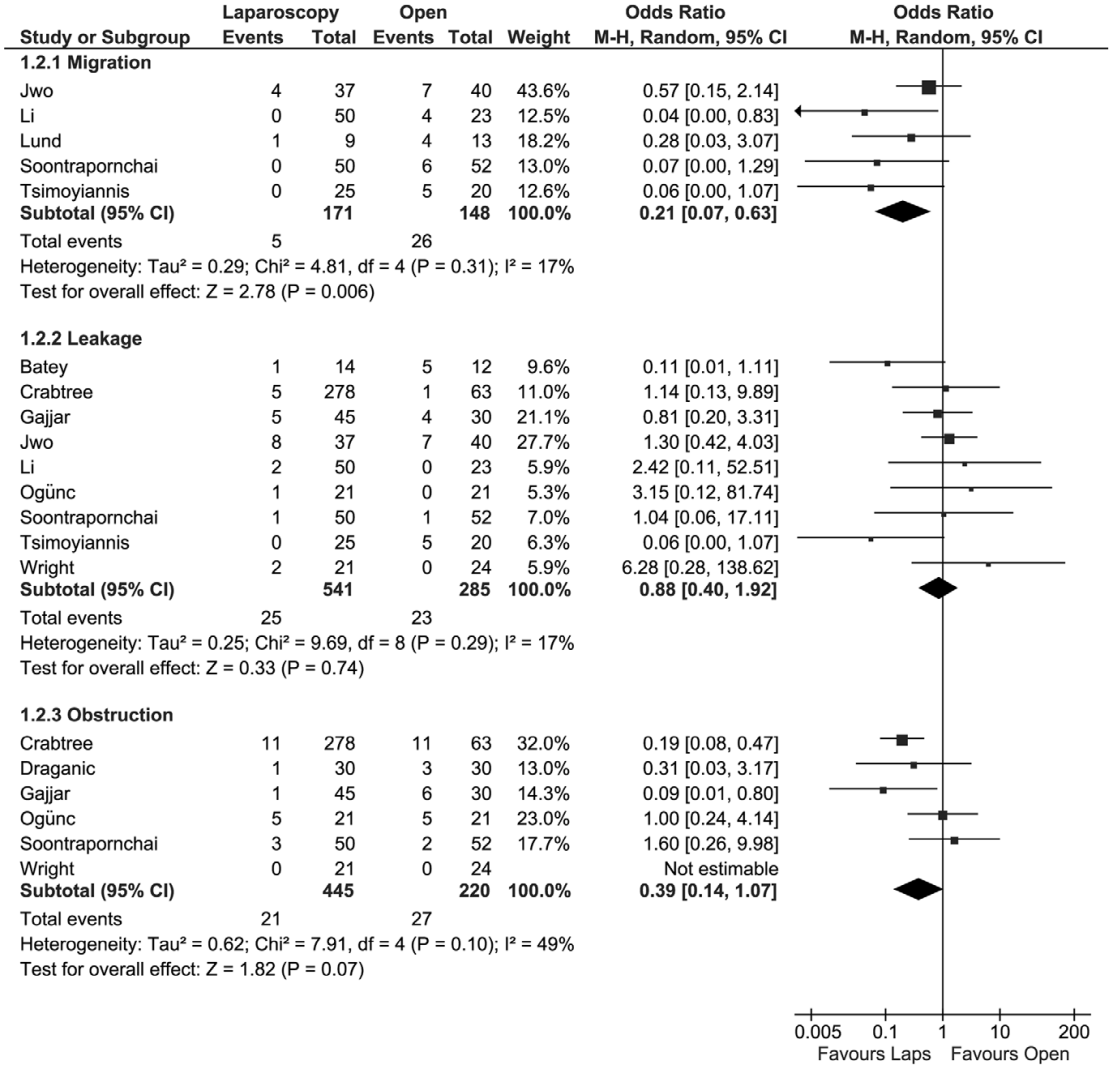
Forest plot. Odds ratios of the incidence of migration, leakage and obstruction, evaluating the statistical difference between laparoscopic and open PD catheter insertion. CI: confidence interval.

The incidence of PD catheter migration was described in five studies [14], [30], [34], [38], [39], with a total of 319 patients, and were used to perform a meta-analysis. Migration occurred statistically significant less frequent in the laparoscopic group (OR 0.21, 95% CI 0.07 to 0.63; P = 0.006). With nine studies [14], [30]–[35], [37]–[39], with a total of 826 patients, the pooled incidence of leakage was calculated. There is no statistically significant difference between the two treatment groups (OR 0.88, 95% CI 0.40 to 1.92; p = 0.74). The incidence of obstructed/dysfunctioning catheters was reported for 665 patients in six studies [31]–[36] and was used for meta-analysis. There was a borderline statistically significant difference in favour of the laparoscopic group in this respect between the two treatment methods (OR 0.39, 95% CI 0.14 to 1.07; P = 0.07) (figure 4).

### Interventional Outcome (Surgical Intervention/Catheter Revision and Removal)

**Figure 5:**
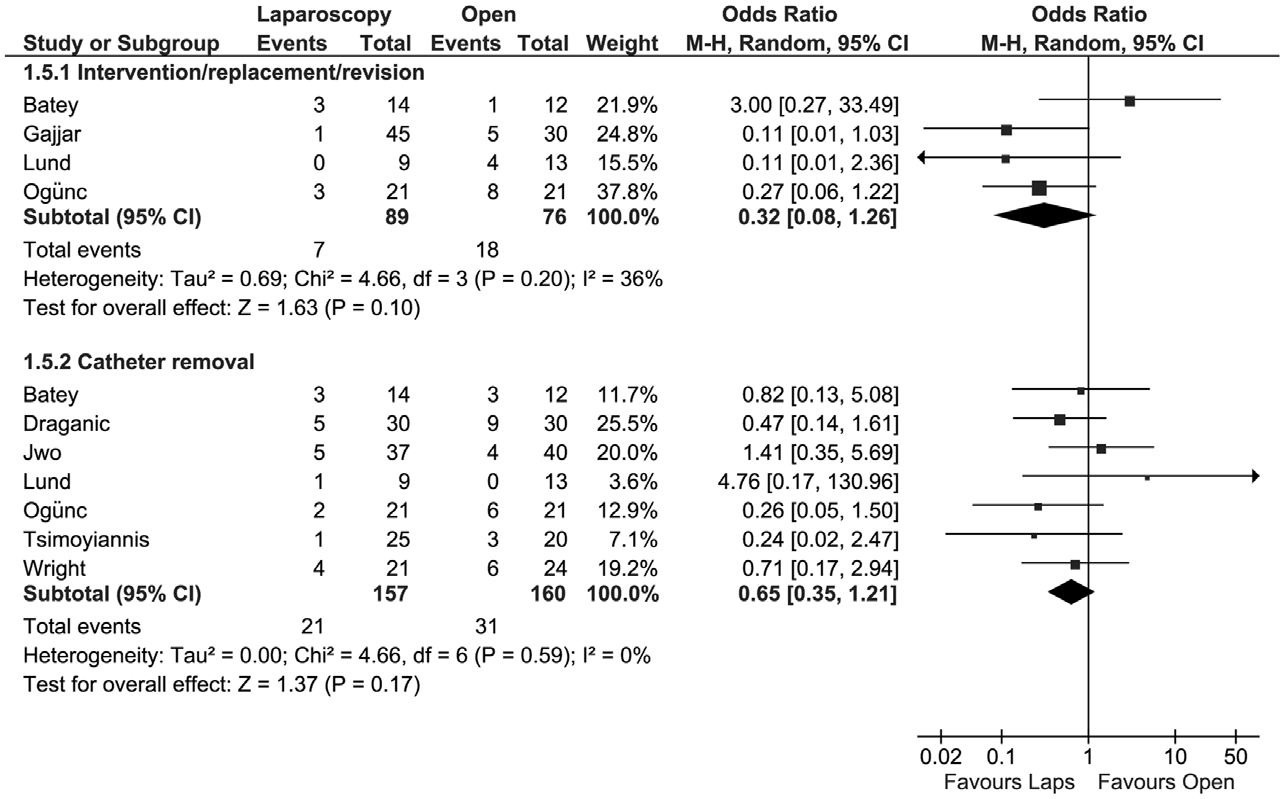
Forest plot. Odds ratio of the incidence of intervention/revision and catheter removal, evaluating the statistical difference between laparoscopic and open PD catheter insertion. CI: confidence interval.

The need for a surgical intervention or catheter revision was described in four studies [32], [35], [37], [38], with a total of 165 patients. After meta-analysis, the need for an intervention showed no difference between groups (OR 0.32, CI 0.08 to 1.26; P = 0.10) The removal of PD catheters as mentioned above was investigated in seven studies [14], [30], [31], [35]–[38], including a total of 317 patients. The meta-analysis showed no statistically significant difference between the two groups. (OR 0.65, 95% CI 0.35 to 1.21; P = 0.17) (figure 5).

### Overall Catheter Survival, Year 1 and 2

**Figure 6:**
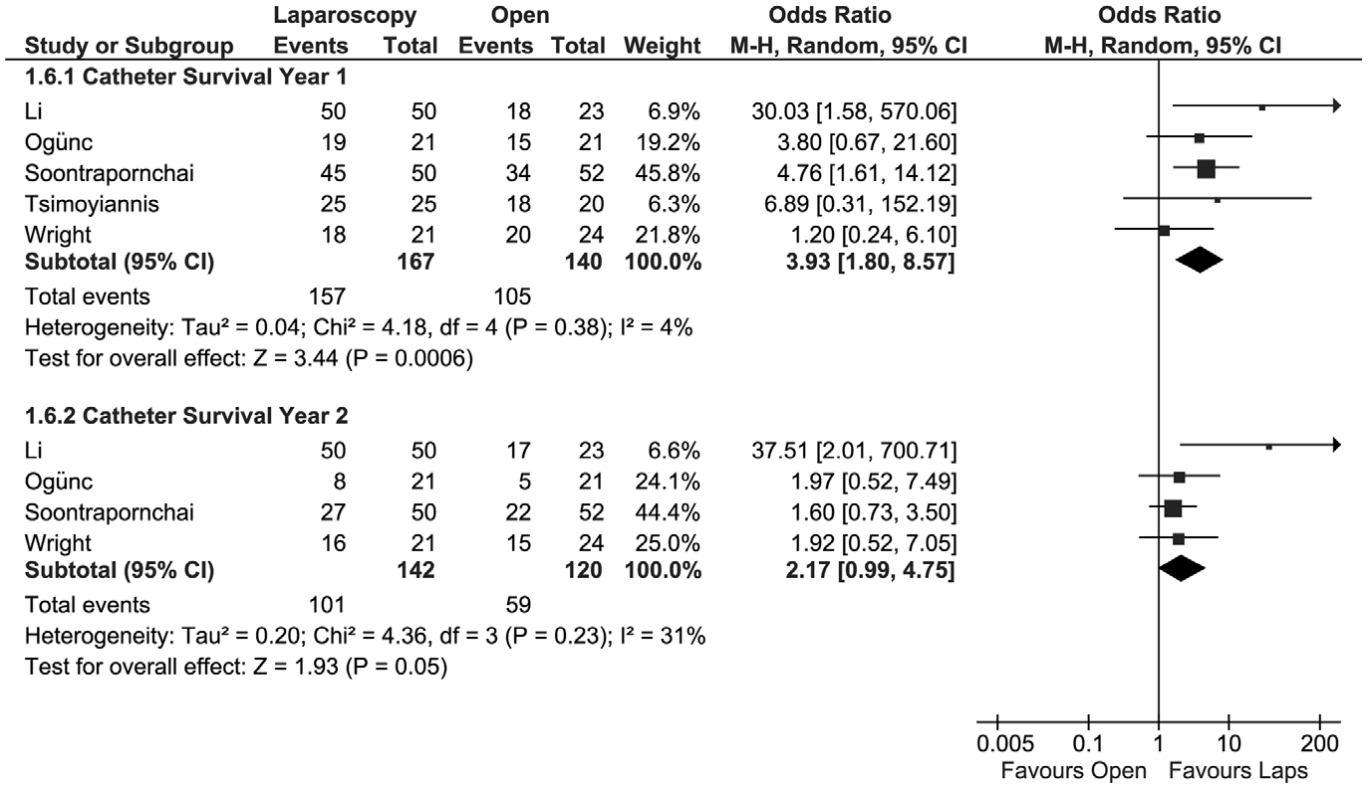
Forest plot. Odds ratios of the catheter survival, at one year and two years after insertion, evaluating the statistical difference between laparoscopic and open PD catheter insertion. CI: confidence interval.

The probability of catheter survival at one year postoperatively was investigated in five studies [30], [31], [34], [35], [39], with a total of 307 patients. The 1-year survival of the catheters was statistically significant higher in the laparoscopic group (OR 3.93, 95% CI 1.80 to 8.57; P = 0.0006) The chance of catheter survival at two years postoperatively was described for 262 patients in four studies [31], [34], [35], [39]. There was a borderline statistically significant difference in catheter survival at this time point (OR 2.17, CI 0.99 to 4.75; P = 0.05) (figure 6).

**Figure 7:**
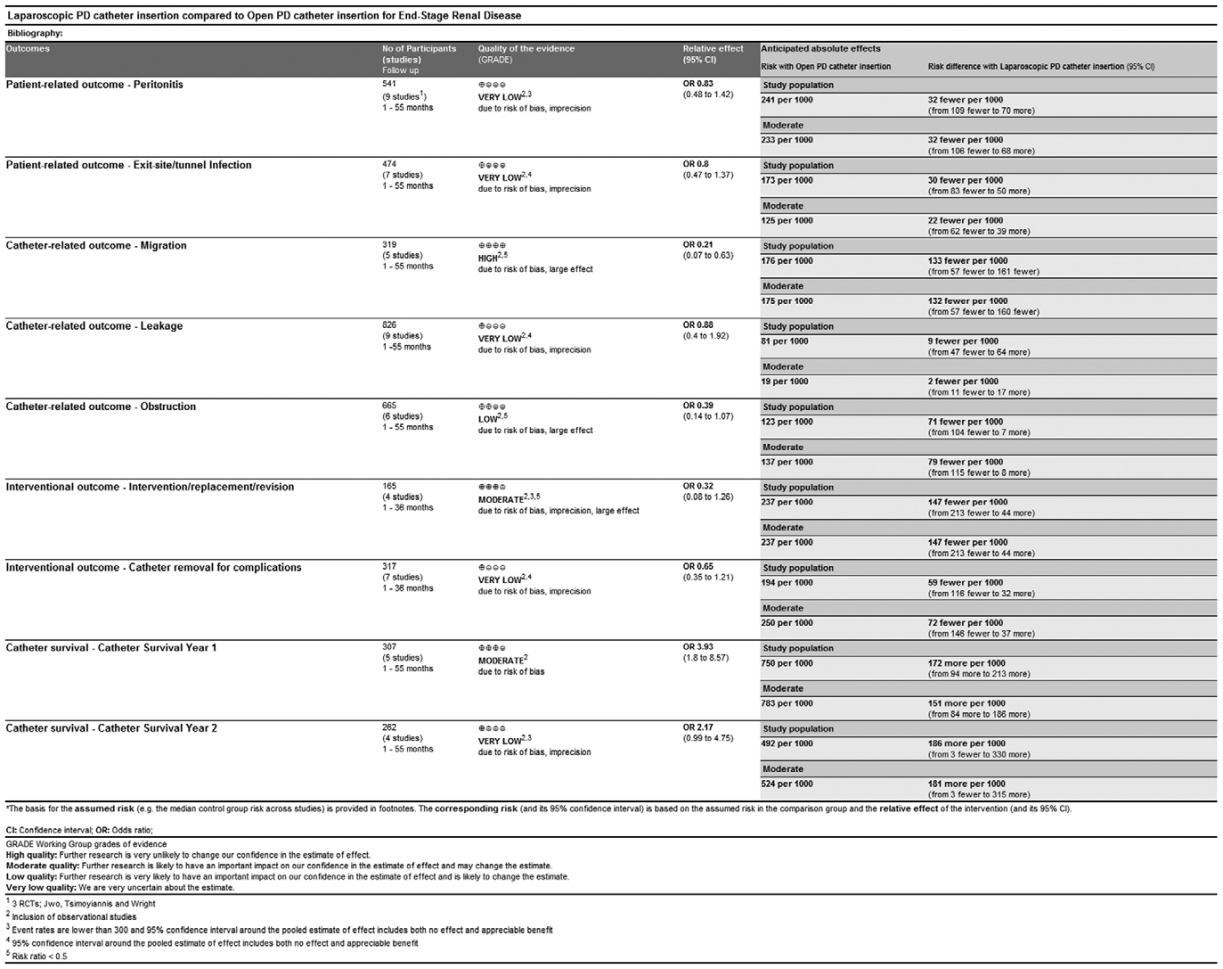
Summery of findings table generated by the GRADE tool.

The quality of evidence of each study and outcome measure are presented as a summary of findings in figure 7. In this figure, the risk differences are presented, using which the numbers needed to treat (NNT) can be derived. For the statistically significant different outcome measures, the NNT are 8 (migration) and 6 (catheter survival year 1). Furthermore, as stated in the methods section, the quality of the RCTs was assessed by the Higgins-classification. No studies were excluded based on this classification. Sensitivity analysis, by removing each study separately, did not change results significantly, except for obstruction (when Ögünç [35] and/or Soontrapornchai [34] were excluded, respectively P = 0.03 and P = 0.01, cumulative P<0.0001) and catheter intervention/replacement/revision (when Batey [37] was excluded, P = 0.004). Additionally, sensitivity analysis was performed per type of study (RCT versus cohort) and no differences were found.

## Discussion

This systematic review and meta-analysis reveals that the laparoscopic PD catheter insertion technique is to be preferred over the conventional open technique. Catheter survival at one year is higher in the laparoscopic group and the incidence of catheter migration is lower in this group. Furthermore, laparoscopic insertion of the PD-catheters assumingly would result in higher patient comfort, lower hospital costs and better overall PD results.

Recently, a similar meta-analysis was published by Xie et al. [24] of which the conclusion is that laparoscopic catheter placement has no superiority to open surgery. However, the authors included two studies that assessed a different technique (peritoneoscopic and percutaneous insertion) and studies including pediatric patients. In our opinion, those studies do not comply with the inclusion criteria that should be used for a meta-analysis regarding this specific topic, being aware of possible selection bias, and therefore potentially a false conclusion is drawn by the authors. In addition, the papers of Lund and Li [38], [39], are not included at all.

Large case series reported no difference in the incidence of peritonitis when using the open insertion technique (2.9–31%) [41]–[44] or the laparoscopic technique (2.5–31%) [15], [45], [46]. The pooled data in this meta-analysis also shows no significant difference in the incidence of peritonitis in agreement with these studies, but there seems to be an overall trend in favour of laparoscopy. The variety in peritonitis incidence in different reports may partly be due to a different antibiotic (AB) prophylaxis regimen used. There is no consensus about which AB to administer and when it should be given to prevent peritonitis. The type of AB used, may influence the incidence of peritonitis [47]. Five studies [30], [34], [36], [38], [39] in our analysis made no mention of (specific) antibiotic prophylaxis, five studies [14], [32], [33], [35], [37] reported the use of cefazolin and one study [31] the use of vancomycin. However, Gadallah [48] reported in a large RCT that the use of 1 g vancomycin preoperatively significantly reduced the risk of developing peritonitis in comparison with 1 g cefazolin and no antibiotic at all. International guidelines state that the use of vancomycin is to be preferred [49].

The incidence of exit-site/tunnel infections does not differ between the laparoscopic and open insertion technique. In all cases, the PD catheter was subcutaneously tunnelled, which is thought to reduce the incidence of exit-site infections, regardless of the insertion technique [50], [51]. The literature, not analyzed in this meta-analysis, suggests a higher incidence of exit-site infections in the open group (6.3–41% [41]–[44]) versus the laparoscopic group (2.5–18% [15], [45]). The time to start the actual PD after catheter insertion may be a possible confounder regarding this issue. Authors of some of the studies included in this analysis favour immediate PD start [30], [35] where others suggest a waiting period of 3 to 5 days [36] or two weeks [31]–[34]. Two studies [35], [36] started PD 1 week earlier in the laparoscopic group than in the open group. Therefore, a definite conclusion is not possible to be drawn. Currently, Ranganathan [52] is performing a randomized controlled trial to determine what the most appropriate time to start PD after catheter insertion might be. The correlation between exit-site infections and peritonitis remains to be elucidated.

One might reason that the influence of the surgical insertion technique on migration is different in the early phase as compared to late phase postoperatively. A subgroup analysis on this issue was desired, but there was insufficient data to perform such an analysis.

Migration is reported in case-series in 1.3–5.4% of the laparoscopically inserted PD catheters [15], [27], [45] and in 7.6–17.1% when using the open technique [41], [44], [53]. A possible advantage of the laparoscopic insertion technique might be the ability to fixate the catheter to the ventral abdominal wall. Jwo, Li, Lund, Soontrapornchai and Tsimoyiannis [14], [30], [34], [38], [39] accurately described the incidence of migration. Li, Soontrapornchai and Tsimoyiannis used a fixation technique in the laparoscopic group; they reported no migration. The overall effectiveness of laparoscopic insertion to prevent catheter migration seems clear, but the benefit of catheter fixation is still under investigation. Ashegh et al. [15] reported 1.3% migration without fixation of the catheter tip and Lo et al. [27] 5.4% with fixation during laparoscopic insertion. Chen et al. [54] used a fixation technique in the open approach and reported no migration. Complication rates are reported to be comparable in case-series using fixation and no fixation [15], [27], [54]. Good clinical trials comparing fixation with no fixation are not available in literature. Besides the suture technique, rectus sheath tunneling might also contribute to a lower migration rate. Soontrapornchai, Ögünç and Crabtree [33]–[35] used this technique, but only Soontrapornchai reported the migration rate accurately and could be included for analysis.

Different types of catheters are used in the studies included in this analysis. This may bias the results of catheter obstruction/dysfunction. Also, the use of either a coiled or a straight catheter might influence the results. Swartz et al. have suggested that the use of coiled catheters reduces the incidence of catheter dysfunction [55]. The literature, not analyzed in the meta-analysis, does not show consensus at this point. Johnson et al. [41] performed a RCT to evaluate the use of a coiled and a straight catheter and reported a significantly higher one-year survival when using a straight catheter (64% vs. 75% respectively). However, Nielsen et al. [56] also performed a RCT comparing coiled and straight catheters, and reported a significantly higher one year survival of coiled catheters (77% vs. 36% respectively). Johnson inserted the catheters using the open method, where Nielsen used a percutaneous technique. The importance of the type of catheters inserted laparoscopically remains unknown at this point. The ideal type of catheter may depend on the operative insertion technique.

The incidence of dialysate leakage is not significantly different between the laparoscopic and open insertion technique. As with the incidence of peritonitis, the time to start PD may also influence the occurrence of leakage. Starting PD shortly after insertion might cause an increased percentage of persistent leakage, for not allowing the peritoneum to heal properly. A possible confounder might be the number of cuffs on the catheter used. Most studies in this meta-analysis used a double-cuffed catheter, Gajjar [32] used single cuffed as well as double cuffed catheters. In the literature, comparative studies indicate no difference in outcome between double and single cuffed catheters [57], [58]. However, these studies did not use the laparoscopic insertion technique. It is possible that the number of cuffs used influences the incidence of leakage when using the laparoscopic insertion technique, but not when using the open technique. However, this meta-analysis and review cannot give a solution to this problem.

In case of dysfunctioning catheters, a laparoscopic revision was successfully performed in most cases. Catheter insertion via the open technique required more interventions or revisions, although not significant (P = 0.07), which may lead to a lower patient comfort and higher costs. The results might be biased, because not all studies reported whether an intervention was performed in case of a dysfunctioning catheter. A cost-benefit analysis is recommended at this point.

Most importantly, PD catheters that were inserted using the laparoscopic technique have been demonstrated to have a significantly higher 1 year survival. Remarkably, the 2-year catheter survival is only borderline significantly different between the groups. This can be attributed to the fact that Tsimoyiannis et al. is not included in the analysis, because of a shorter follow-up than 2 years, resulting in a smaller number of analyzed patients.

Most studies analyzed in this meta-analysis, reported the survival in percentages, where a Kaplan-Meier curve is to be preferred. The reporting of proportions might have led to inaccurate survival data.

Although the incidence of most complications, except for catheter migration, individually is not significantly different between laparoscopic and open insertion, all studies combined show that laparoscopically inserted catheters tend to enable better and prolonged PD.

### Limitations

In order to include sufficient patient data to draw solid conclusions, both observational and intervention studies were included. This might have lead to selection bias. In our opinion, it was more important to have a larger number of included patients than the inclusion of interventional studies only, despite the fact that cohort studies are more prone to possible bias. Furthermore, the observational studies support the findings of the RCTs, as we confirmed in our sensitivity analysis. According to the assessment using the GRADE tool, we conclude that the evidence of each individual included study varies from very low to high. However, the highest level of evidence was found for the outcome measures that showed significant differences in our analysis. One other possible limitation is that the analysis might be biased because of difference in the individual experience of the operating surgeons. Furthermore, the procedures are not always carried out by one surgeon only. Possible downside of the analyzed studies is the small number of patients in both intervention arms for some outcome measures. Small patient groups increase the chance of getting smaller or larger differences based on random chance. Despite the statistical homogeneity, some outcome measures appear to be clinically heterogeneous. This might be caused by possible center bias.

Despite these limitations, this meta-analysis is the first step in giving a definite answer as to which procedure of the two (laparoscopic or open insertion technique) might be the better procedure for reducing complications and better PD catheter survival. This systematic review and meta-analysis reveals the potential benefits of laparoscopic PD-catheter insertion. In order to be able to evaluate the true value of laparoscopy in PD-catheter insertion, a large randomized controlled trial is recommended [59].
